# Cyclic dual latent discovery for improved blood glucose prediction through patient–provider interaction modeling: a prediction study

**DOI:** 10.12771/emj.2025.00332

**Published:** 2025-04-15

**Authors:** Suyeon Park, Seoyoung Kim, Dohyoung Rim

**Affiliations:** 1 Ewha Womans University College of Medicine, Seoul, Korea; 2 Rowan Corporation, Seoul, Korea

**Keywords:** Blood glucose, Biometry, Deep learning, Diabetes mellitus, Hyperglycemia, United States

## Abstract

**Purpose:**

Accurate prediction of blood glucose variability is crucial for effective diabetes management, as both hypoglycemia and hyperglycemia are associated with increased morbidity and mortality. However, conventional predictive models rely primarily on patient-specific biometric data, often neglecting the influence of patient–provider interactions, which can significantly impact outcomes. This study introduces Cyclic Dual Latent Discovery (CDLD), a deep learning framework that explicitly models patient–provider interactions to improve prediction of blood glucose levels. By leveraging a real-world intensive care unit (ICU) dataset, the model captures latent attributes of both patients and providers, thus improving forecasting accuracy.

**Methods:**

ICU patient records were obtained from the MIMIC-IV v3.0 critical care database, including approximately 5,014 instances of patient–provider interaction. The CDLD model uses a cyclic training mechanism that alternately updates patient and provider latent representations to optimize predictive performance. During preprocessing, all numeric features were normalized, and extreme glucose values were capped at 500 mg/dL to mitigate the effect of outliers.

**Results:**

CDLD outperformed conventional models, achieving a root mean square error of 0.0852 on the validation set and 0.0899 on the test set, which indicates improved generalization. The model effectively captured latent patient–provider interaction patterns, yielding more accurate glucose variability predictions than baseline approaches.

**Conclusion:**

Integrating patient–provider interaction modeling into predictive frameworks can increase blood glucose prediction accuracy. The CDLD model offers a novel approach to diabetes management, potentially paving the way for artificial intelligence-driven personalized treatment strategies.

## Introduction

### Background

Accurate prediction of blood glucose variability is crucial for effective diabetes management and the prevention of acute complications. In the management of diabetes, predicting blood glucose levels can help prevent hypoglycemia and hyperglycemia [1]. Forecasting drops in glucose levels allows individuals to take preventive measures before they experience dizziness, confusion, or loss of consciousness. Similarly, forecasting hyperglycemic episodes enables timely adjustments in clinical treatments. Early detection and prediction of rising glucose levels aid in adjusting insulin dosages and other clinical treatments [2]. Blood sugar prediction facilitates proactive management, reducing health risks caused by consistently high blood glucose levels, such as heart stroke, nephropathy, neuropathy, retinopathy, and foot problems [3]. Moreover, predictive tools can alleviate patients’ anxiety about sudden blood sugar fluctuations. Providing early warnings and reducing the burden of constant monitoring can offer greater flexibility in settings such as the work environment.

Patient–provider interactions influence factors such as medication adherence, dietary choices, and lifestyle modifications [4]. A substantial body of research indicates that variations among healthcare providers may significantly contribute to differences in the prognoses of patients with similar initial conditions [5]. However, traditional prediction models rely primarily on patient-specific biometric indicators and often overlook external influences, including interactions with physicians and other healthcare providers.

To address this gap, our study applies the previously proposed Cyclic Dual Latent Discovery (CDLD) model to the task of prediction based on medical data that include information regarding healthcare providers, leveraging the Cyclic Dual Latent Discovery (CDLD) model [6]. CDLD is a deep learning framework designed to capture the interrelationship between 2 entities to predict the result of their interaction. In contrast to previous artificial intelligence (AI) studies that have not fully incorporated patient–healthcare provider interactions, the CDLD model integrates these relationships to establish a more comprehensive and clinically significant predictive framework for biological parameters such as blood glucose levels.

### Objectives

Our work aimed to bridge the gap between individual biometric predictors and a more holistic interaction-based approach. Specifically, we trained the CDLD model to predict the blood glucose levels of patients in the intensive care unit (ICU). The CDLD model discovered latent triats for both patients and providers and used them to predict final blood glucose levels, with predictive accuracy assessed via RMSE on the held-out test set.

## Methods

### Ethics statement

This study was exempt from institutional review board approval because it used only de-identified, publicly available data (from the MIMIC-IV dataset) [7].

To access this database, we gained credentialed access by completing the “CITI Data or Specimens Only Research” training course and signing the “PhysioNet Credentialed Health Data Use Agreement 1.5.0.” Personal patient identifiers were removed in accordance with Health Insurance Portability and Accountability Act regulations, and random integer IDs were assigned instead. Free text data were checked for protected health information and de-identified if needed.

### Study design

This is a retrospective cohort study designed to predict glucose levels based on patient–provider relationships. It was described according to the TRIPOD-AI reporting guidelines for articles on deep learning in the medical field (development or prediction), available at https://www.tripod-statement.org/.

### Setting

Dates and times were randomly shifted, with a single date shift applied for each patient to maintain internal consistency and different shifts for distinct patients to ensure de-identification.

### Participants

The study participants were patients admitted to either the emergency department or the ICU between 2008 and 2019 at Beth Israel Deaconess Medical Center, Boston, Massachusetts, United States. Patients were excluded if they were younger than 18 years at their first visit or if they were on an established list of vulnerable groups requiring enhanced protection.

### Data source

The data used in this study were obtained from PhysioNet, specifically from the MIMIC-IV project [7]. After restructuring the data for easier analysis—including de-normalizing, removing audit trails, and reorganizing—de-identification was conducted to maintain patient privacy.

The MIMIC-IV v3.0 dataset was grouped into 2 modules: hosp and icu. The hosp module is sourced from the hospital-wide electronic health record, and the icu module is derived from MetaVision, the in-ICU clinical information system. The full database includes a total of 364,627 individuals who experienced 546,028 unique hospitalizations and 94,458 unique ICU stays.

Within the hosp module, the records from the 546,028 hospitalizations correspond to 223,452 unique individuals. The dataframes include patient demographics (patients), hospitalizations (admissions), and intra-hospital transfers (transfers); laboratory measurements (labevents, d_labitems); microbiology cultures (microbiologyevents, d_micro); provider orders (poe, poe_detail); medication administration (emar, emar_detail); medication prescription (prescriptions, pharmacy); hospital billing information (diagnoses_icd, d_icd_diagnoses, procedures_icd, d_icd_procedures, hcpcsevents, d_hcpcs, drgcodes); online medical record data (omr); and service-related information (services). Provider information was also included in the provider table, with a deidentified character string present in the provider_id column.

The icu module contains data from 94,458 unique ICU stays experienced by 65,366 unique individuals. The dataframes include intravenous and fluid inputs (inputevents), ingredients for these inputs (ingredientevents), patient outputs (outputevents), procedures (procedureevents), information documented as a date or time (datetimeevents), and other charted information (chartevents). All events tables contain a stay_id column, enabling identification of the ICU patient associated with the stay, as well as an itemid column to identify the concept documented in d_items. The caregiver table, referencing the care provider who collected the data as a deidentified integer (caregiver_id), was also included and linked to all event tables.

From this database, we extracted a subset that suited our objectives. The dataset consisted of 5,014 patients. The sample included 2,696 male and 2,304 female patients, with an average age of 63.173. Individual provider identification codes were used to examine the impact of the patient–provider relationship on blood glucose levels.

We focused on patients who had at least one abnormal blood glucose reading during their hospital stay. The highest blood glucose level during the stay was extracted as a feature of the included patients, and the last measured blood glucose level was extracted as a feature representing the result of the interaction (object of prediction) to evaluate the variability of blood glucose based on ICU management. Consequently, the patient entity included an identification code (subject_id and hadm_id), 8 binary features (corresponding to age and gender), and one numerical feature (the highest blood glucose level during the stay). The provider entity consisted solely of an identification code. One numerical feature (the last measured blood glucose level) was used as the prediction target.

**Fig. 1.**
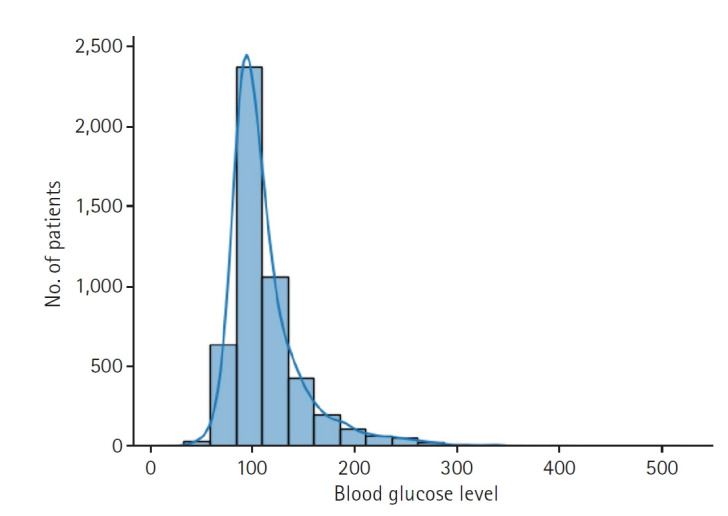
Distribution of the last measured blood glucose level, the prediction target of this study.

The dataset used in the study includes 5,001 glucose level measurements from 2,551 patients. Some data were excluded for training efficiency. Overall, 80% of the data were used for training, 10% for validation, and the remaining 10% for testing (Fig. 1).

### Data preprocessing

To adapt the MIMIC-IV dataset for input into the CDLD model, we performed a series of preprocessing steps to appropriately structure patient and provider data. The dataset was organized into 3 main components as follows: first, patient data (user), which included demographic and clinical features such as gender, age (transformed into 6 categorical variables), and peak blood glucose levels; second, provider data (item), represented by de-identified provider IDs; and third, interaction data (rating), capturing patient–provider interactions, with the final recorded blood glucose level as the target variable.

We extracted patient features from the “admissions” and “patients” datasets. Certain attributes, such as race, language, type of insurance, and admission details, were excluded due to cost concerns and because they only minimally affect changes in blood glucose levels. Accordingly, the subject_id, hadm_id, gender, and age of each patient were retained as patient features.

By examining the “labevents” dataset, we selected patients with abnormal blood glucose levels (≥125 mg/dL) because these patients are closely monitored and regulated. In the ICU, continuous blood glucose level regulation is considered standard [8,9]. Thus, by extracting the highest and the last blood glucose levels for these patients, we can capture the interaction between medical providers and patients in regulating blood glucose levels.

Beyond clinical considerations, we preprocessed the datasets to improve the accuracy and efficiency of CDLD processing. Rather than using age directly, we categorized patient ages into 10-year intervals, converting these into 6 Boolean features. Similarly, gender was represented with 2 Boolean features. All other numerical features were scaled to a range between 0 and 1 to improve processing accuracy. Because outliers with blood glucose levels over 500 mg/dL influenced the overall standardized values and hindered effective standardization, data points exceeding 500 were assigned a value of 500. As a result, the glucose levels used in the study were limited to a range of 125 to 500 mg/dL and normalized to a numerical range of 0 to 1. Predictions of glucose level were also expressed as normalized values.

For provider data, only the “provider_id” was available from the “poe” dataset. This alphanumeric code was assigned to physicians in the hospital to differentiate them without revealing personal information. To improve the accuracy of CDLD processing, these codes were converted into numerical values. The dataframes used in the study are presented in Supplement 1.

When extracting the provider for each patient during training of the CDLD model, the most frequently recorded provider during the hospital stay was regarded as the interacting provider because no information was available about the providers’ clinical hierarchy. We thus assumed that the provider with the most interactions was the one who had the most influence. Although detailed information about physicians was not available and may have been insufficient to reflect each latent feature, we assumed that the CDLD model could appropriately discover the latent features of each provider_id through cyclic training.

### Outcome variables

The primary outcome variable was the blood glucose level measured at discharge. Patient variables included sex, age, and peak blood glucose level during hospitalization, while provider was considered as an additional variable. These variables were treated as latent traits.

### Study size

All target patient data were extracted from the database; thus, no sample size estimation was performed. For the CDLD deep learning model, a sample of 5,001 participants was deemed sufficient for evaluation based on the estimation power of the model, which delivers performance regardless of the case number.

### Deep learning models

CDLD is a recently proposed modeling approach that employs 2 neural networks in a cyclic training loop to uncover hidden (latent) traits of 2 interacting entities from their interaction data. The principle is that each interaction between 2 entities (e.g., a user and an item in a recommender system) contains intrinsic information about both. In the CDLD architecture, one network learns a representation for entity A (e.g., the patient), while a second network learns a representation for entity B (e.g., the provider). These networks are then trained alternately in a cyclic fashion. Through this iterative refinement process, each network’s output informs the training of the other, allowing the model to capture complex non-linear relationships more effectively than traditional single-pass or linear models.

In our implementation, the 2 entity types in CDLD are patients (analogous to users) and healthcare providers (analogous to items). The model discovers latent attributes for each patient and provider based on their clinical encounter data, and it cyclically updates these representations to predict the outcome of their interaction—in this case, the patient’s post-care blood glucose level. By leveraging CDLD in this manner, we directly incorporate the patient–provider relationship into the prediction framework, yielding a more comprehensive model than one based only on patient features.

To evaluate the effectiveness of the CDLD model in predicting blood glucose variability, we conducted a series of experiments using patient–provider interaction data. The experimental setup involved data preprocessing, model training, and performance evaluation.

We implemented the CDLD model to learn latent representations for patients and providers. As described above, the model consists of 2 neural sub-networks (patient-latent discoverer and provider-latent discoverer) that are trained alternately in a cyclic fashion; the output of one network helps update the other. Key training hyper-parameters were as follows: a latent vector dimension of 32, a batch size of 1,024, and training for 10 epochs (with 10 sub-epochs per cyclic iteration). We used the Adam optimizer and optimized a mean square error (MSE) loss, with root mean square error (RMSE) used as the evaluation metric. To improve generalization, we applied data augmentation by adding small random noise (magnitude 0.1) to input features, effectively increasing the training dataset size fivefold.

### Evaluation metrics

The data were split into training (80%), validation (10%), and test (10%) sets. Model performance was evaluated primarily based on RMSE for blood glucose predictions. RMSE was computed for the validation and test sets to assess generalization, as well as for the training set to monitor potential overfitting.

### Statistical methods

No statistical tests were performed, aside from error measurement using RMSE.

## Results

**Fig. 2.**
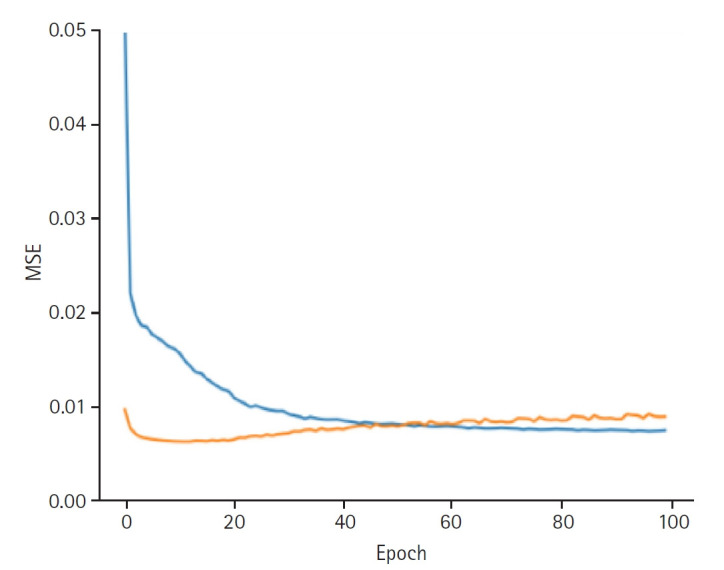
Graphs depicting the results for model performance when discovering the patient latent. The blue line represents the “train loss,” while the orange line denotes the “valid loss.” The x-axis is labeled “epoch,” indicating the number of training iterations, and the y-axis is labeled mean squared error (“MSE”), which measures the prediction error of the model.

**Fig. 3.**
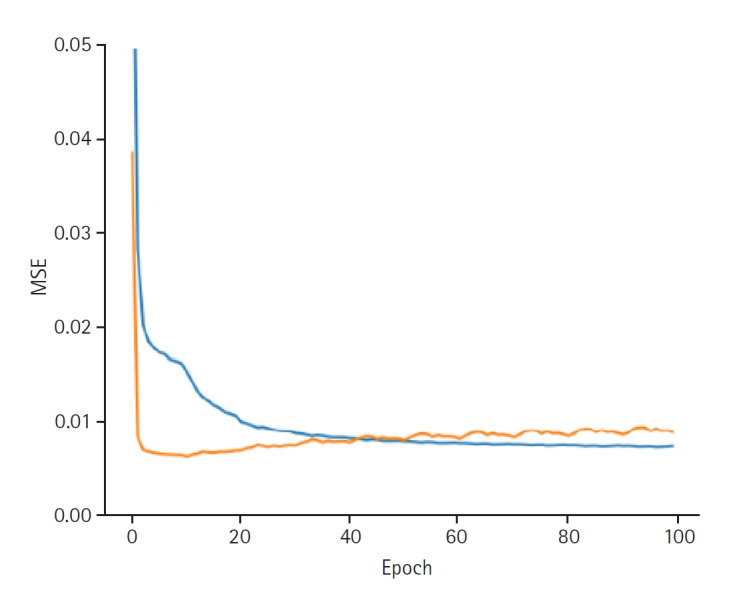
Graphs depicting the results for model performance when discovering the provider latent. The blue line represents the “train loss,” while the orange line denotes the “valid loss.” The x-axis is labeled “epoch,” indicating the number of training iterations, and the y-axis is labeled mean squared error (“MSE”), which measures the prediction error of the model.

When discovering the patient latent, the model achieved a training loss of 0.0066 and a validation loss of 0.0089. The RMSE for the training set was 0.0813, and the validation RMSE was 0.0941 (Fig. 2). When discovering the provider latent, the model exhibited a training loss of 0.0066 and a validation loss of 0.0088. The RMSE for the training set was 0.0810, while the validation RMSE was 0.0937 (Fig. 3).

**Fig. 4.**
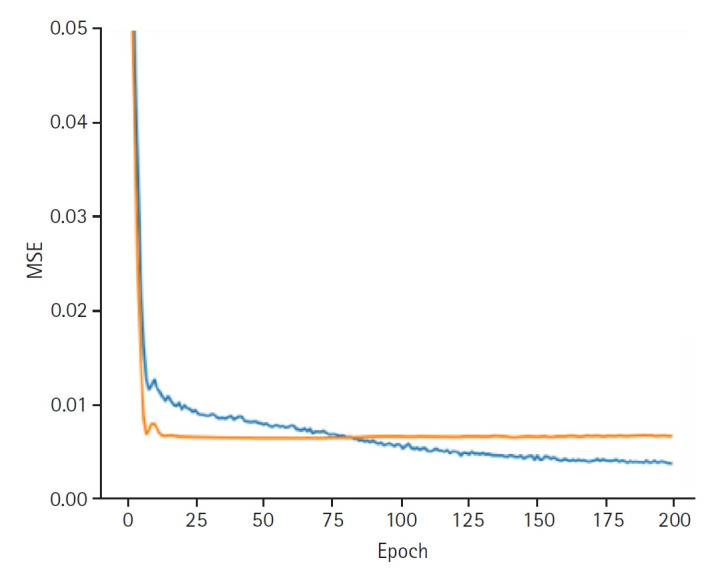
Graphs depicting the results for model performance when predicting the last measured glucose level. The blue line represents the “train loss,” while the orange line denotes the “valid loss.” The x-axis is labeled “epoch,” indicating the number of training iterations, and the y-axis is labeled mean squared error (“MSE”), which measures the prediction error of the model.

In synthesizing these latent representations to predict blood glucose variability, the model achieved a training loss of 0.0037 and a validation loss of 0.0066. The training set RMSE was 0.0336, the validation RMSE was 0.0852, and the test RMSE was 0.0898 (Fig. 4).

These values are sufficiently low, indicating strong predictive accuracy of the CDLD model regarding blood glucose variability.

## Discussion

### Key results

The final predictor that synthesized the latent representations achieved a test RMSE of 0.0898, indicating strong predictive performance. These values are sufficiently low to indicate that the model achieved a balanced and practically significant level of predictive accuracy. Although the slightly higher validation RMSE suggests minor overfitting, it remains within an acceptable range, supporting the model’s generalizability.

### Interpretation

The results demonstrate that the CDLD model effectively captured patient–provider interactions and provided accurate predictions for blood glucose variability. This confirms its viability for enhancing diabetes management strategies based on patient-provider interactions. The findings indicate a well-fitted model that maintains good performance while exhibiting a modest increase in error on unseen data.

Moreover, the CDLD framework handles data with a non-linear structure, overcoming the constraints of single entity-based datasets. This capability enables applications beyond individual predictions, such as evaluating healthcare provider performance and improving patient management strategies. By incorporating provider-related variables, the model can assist in assessing medical quality and optimizing treatment methodologies.

### Comparison with previous studies

Traditional blood glucose prediction models predominantly utilize biometric data from individual patients [4], which limits their capacity to incorporate external influences such as physician intervention. The Multi-source Irregular Time-Series Transformer (MITST) uses a transformer-based hierarchical model to integrate data and capture temporal dynamics, thereby eliminating manual feature engineering [10]. Evaluated on the eICU database (including 200,859 ICU stays across 208 hospitals) [11], it outperforms the baseline by 1.7% in area under the receiver operating characteristic curve (AUROC) and by 1.8% in area under the precision-recall curve (AUPRC) (P<0.001). For hypoglycemia, it achieves an AUROC of 0.915 and an AUPRC of 0.247 [12]. Huang et al. [13] in 2023 proposed a graph-based hierarchical network to learn from multiple patients’ continuous glucose monitoring data, significantly reducing prediction error by approximately 30% compared to standard models. In critical care, Tang et al. [2] in 2022 reported an RMSE of about 15.8 mg/dL for post-insulin glucose. Outpatient models also perform well; the LSTM of Shao et al. [14] in 2024 achieved an AUROC above 0.97 for hypoglycemia across diabetes types, while the Glu-Ensemble for type 2 diabetes by Han et al. [15] in 2024 improved RMSE without patient-specific calibration. These advanced architectures consistently outperform traditional linear models, improving accuracy and sensitivity regarding glycemic events.

By leveraging entity-to-entity interactions, the CDLD enables a more comprehensive approach, explicitly modeling the relationships between patients and healthcare providers. This advancement facilitates a better understanding of how medical care influences elements of patients’ medical status, such as blood glucose levels, beyond direct physiological factors.

### Limitations

The primary limitation of this study arises from its use of minimal information. The research could not account for various aspects of the healthcare providers, making it nearly impossible to determine which aspects of patient–provider interactions contribute to fluctuations in glucose levels. This limitation could pose challenges in real-world implementations, as providing actionable feedback to healthcare providers based solely on this information would be difficult. Additionally, privacy concerns regarding medical personnel data present potential obstacles for practical application. Addressing these concerns will be crucial for broader adoption in clinical settings.

### Clinical implications

Notably, the CDLD model can predict patients’ glucose levels without requiring detailed information about the provider.

Proper management of patients’ blood glucose levels is crucial, as hyperglycemia or hypoglycemia can adversely impact overall health by causing various complications, particularly among ICU patients [16]. Therefore, thorough monitoring and prediction of blood glucose fluctuations are essential for preventing both hyperglycemia and hypoglycemia [17]. With a more precise understanding of these fluctuations, clinicians can implement targeted interventions and better prepare to manage high-risk patients, ultimately reducing adverse outcomes. In some cases, overly strict glycemic control can be counterproductive; thus, it is essential to manage blood glucose levels carefully [18].

However, because each patient’s condition can vary significantly, tailoring glucose management strategies remains challenging. By incorporating various factors into the CDLD model that affect glucose levels—including data on medical providers, which are often overlooked—clinicians can develop more effective strategies based on accurate predictions. Emerging evidence highlights the importance of personalized treatment for critically ill patients, and integrating diverse factors with precise prediction methods can further enhance these individualized therapies [19].

Predicting changes in glucose levels can also help optimize resource allocation [20]. Serving as an assistive tool for clinical judgment, accurate predictions enable early differentiation between severe and mild cases. This facilitates a more effective distribution of medical resources and personnel, such as ICU beds, ventilators, and intensive monitoring. Early identification of mild cases permits their management in general wards or intermediate care units, preventing unnecessary ICU admissions and reducing bottlenecks. This, in turn, can lower the costs associated with intensive care and contribute to a more sustainable healthcare system.

Predicted blood glucose levels can also support continuous monitoring and early intervention [8]. Detecting fluctuations early and implementing proactive measures can help mitigate the risk of complications, including infections, cardiovascular diseases, and other metabolic disorders. This can lead to improved prognosis and survival among ICU patients.

### Generalizability

The results of this study should be interpreted in the context of the described limitations. However, the modeling approach used can be applied to electronic medical records from other medical institutions without difficulty. Since datasets containing information on healthcare providers are often highly sparse, this feature is expected to be useful for other datasets that lack comprehensive provider-related data.

### Suggestion for further studies

Since deep learning models can assess the influence of each feature after training, we believe that expanding the dataset for CDLD could enable a more in-depth analysis.

### Conclusion

The CDLD model accurately predicted ICU patients’ blood glucose levels by incorporating not only patient-specific data but also healthcare provider information. This proof of concept highlights the value of modeling patient–provider interactions and demonstrates improved predictive performance compared to patient-only models. Future work should further validate this approach and explore how to interpret provider-related factors; nevertheless, our findings suggest that integrating provider interactions can enhance personalized diabetes management in critical care settings.

## Supplementary materials

Supplementary files are available from Harvard Dataverse: https://doi.org/10.7910/DVN/4DTE3N

Supplement 1. Dataframes used in the study. The Cyclic Dual Latent Discovery source code executed is patented and therefore cannot be shared. For further questions, please contact the corresponding author.
